# Malakoplakia with aberrant ALK expression by immunohistochemistry: a case report

**DOI:** 10.1186/s13000-023-01383-z

**Published:** 2023-08-29

**Authors:** Xiao-Ying Zhang, Jun Li, Shui-lian Chen, Ying Li, Hao Wang, Jin-hua He

**Affiliations:** 1 Department of Pathology, Panyu District Central Hospital, Guangzhou, China; 2 Department of Urology, Panyu District Central Hospital, Guangzhou, China; 3 Department of Laboratory Medicine, Panyu District Central Hospital, Guangzhou, China

**Keywords:** ALK, Aberrant expression, Multisystem, Malakoplakia, Michaelis–Gutmann bodies, Liver, Kidney, Colon

## Abstract

**Background:**

Malakoplakia is a rare inflammatory disease of the urogenital tract. There have been no reports of malakoplakia expressing anaplastic lymphoma kinase (ALK) to date. Here, we present one case of malakoplakia with aberrant ALK expression by immunohistochemistry and discuss the clinical significance.

**Case presentation:**

A 65-year-old Chinese woman with a history of diabetes presented with solid masses in the liver and kidney and elevated lesions on the mucosal surface of the colon. Right nephrectomy and partial liver resection were performed. Microscopically, sheets of histiocytes with poor intercellular adhesion were seen, with Michaelis–Gutmann bodies present in both the intracellular and extracellular interstitium. CD10-, CD68-, and CD163-positive cells were present, with Michaelis–Gutmann bodies confirmed by staining with Alcian blue, periodic acid-Schiff (PAS), periodic acid-Schiff with diastase, Von Kossa, and Prussian blue. Aberrant ALK1 and ALK (D5F3) expression was observed in the cytoplasm and nucleus of cells. However, *ALK* gene mutation was not detected by fluorescence in situ hybridization or whole exome next-generation sequencing. NGS revealed nine individual somatic gene mutations: *GOT1L1, GLIS2, SPOUT1, TMEM97, MUC3A, NSD2, SFXN5, ADAD1* and *RAD50*. The significance of the somatic gene mutations detected in this study is not clear, and the relationship between them and malakoplakia cannot be clarified by existing scientific studies. The pathological diagnosis was malakoplakia with aberrant ALK expression by immunohistochemistry. The antibiotics imipenem and vancomycin were started based on the results of drug sensitivity analysis and the patient was subsequently discharged. She experienced no discomfort during 30 months of follow-up.

**Conclusion:**

This is the first reported case of malakoplakia with aberrant ALK expression, it should be differentiated from ALK-positive histiocytosis to avoid misdiagnosis.

**Supplementary Information:**

The online version contains supplementary material available at 10.1186/s13000-023-01383-z.

## Background

Malakoplakia is a rare inflammatory disease caused by the host response to infection by a variety of microorganisms, including *Escherichia coli*, *Klebsiella sp.* and *Acid-fast bacilli sp. *[1]. It was originally described by von Hansemann in 1901, and its morphological characteristics were published by Michaelis and Gutmann 1 year later [2]. It mainly occurs in the urogenital tract, especially the bladder [3], and yellow patch-like lesions are formed on the mucosal surface of the involved organs. Microscopically, it is composed of sheets of cells with characteristic Michaelis–Gutmann (M-G) bodies evident in both the intracellular and extracellular interstitium. Malakoplakia is a benign, self-limiting disease with a generally good prognosis. The expression of anaplastic lymphoma kinase (ALK) in this disease has not been previously reported. Here, we report a case of malakoplakia with aberrant ALK expression by immunohistochemistry and discuss its clinical significance.

## Case presentation

The patient provided consent for publication of this case report.

### Clinical history

**Fig. 1:**
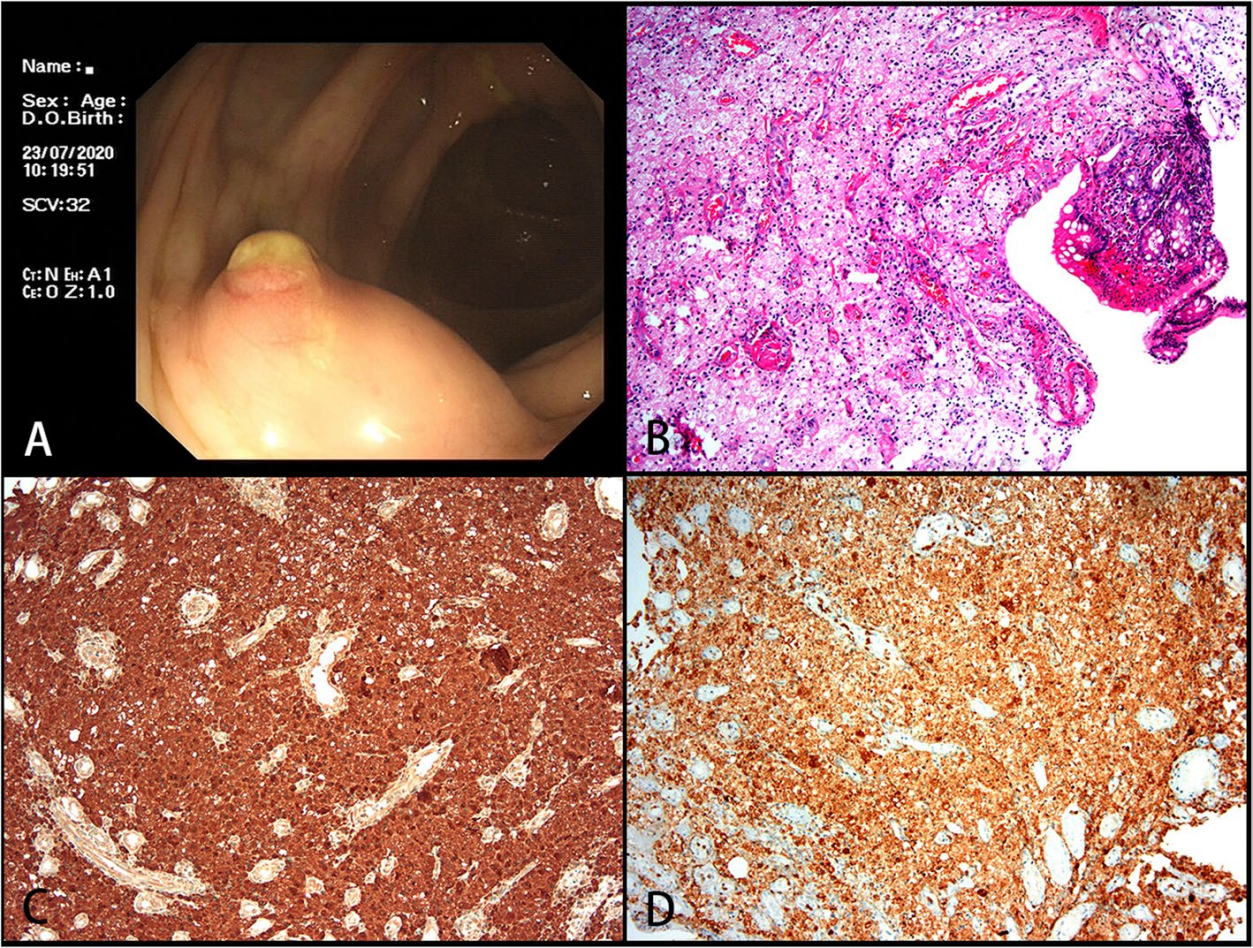
Endoscopic and histological features of malakoplakia in the ascending colon. **A** A hummock-type protrusion in the middle of the ascending colon. Its surface mucosa is smooth and the same color as the surrounding mucosa but with hyperemic mucosa at the top and the edema covered with a white moss-like coating. **B** Disappearance of most of the intestinal mucosa with only small patches of mucosal tissue remaining. The patches are mixed with neutrophils and lymphocytes (H&E, magnification × 100). **C** Histiocytes showing diffuse cytoplasmic CD68 staining (magnification × 100). **D** Histiocytes showing diffuse cytoplasmic CD10 staining (magnification × 100)

**Fig. 2:**
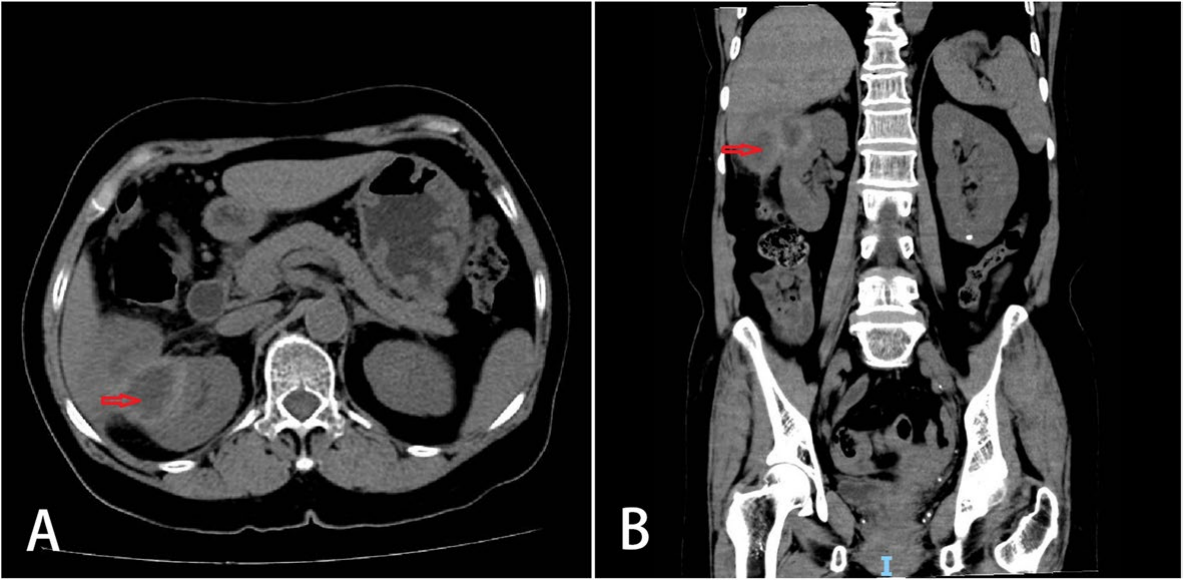
Imaging features of malakoplakia. **A** Computed tomography coronal view showing a circular low-density focus highlighting the renal profile in the upper part of the right kidney (red arrow). The density is uneven, the surrounding fat space is blurred, and the boundary with the right posterior lobe of the liver and the hepatic curvature of the colon is not clear. **B** Sagittal computed tomography showing the lesion was gourd-shaped, with low density in the center and slightly higher density in the periphery (red arrow)

A 65-year-old Chinese woman was admitted with right lumbar and abdominal pain of 1 month’s duration and intermittent fever. She had a long history of diabetes that was well controlled. There were no positive clinical signs except percussive pain in the right renal area. Colonoscopy revealed a 1.6-cm hummock-shaped protrusion in the middle of the ascending colon with a smooth mucosal surface that was the same color as the surrounding mucosa; its texture was soft when touched with biopsy forceps. An abscess (0.4 cm) was observed on one side of the protruding lesion, with local mucosal hyperemia and edema covered with a white moss-like coating (Fig. 1a). A small biopsy of intestinal mucosal tissue was taken by forceps from the lesion and sent for pathological examination. Computed tomography showed a soft tissue shadow measuring 59 × 58 × 45 mm at the lateral margin of the middle and upper parts of the right kidney, with high and low mixed density on plain scans, uneven ring enhancement on enhanced scans, an irregular shape, blurred surrounding fat space, poor demarcation between the lesion and the right posterior lobe of the liver and the hepatic curvature of the colon, and a slightly thickened adjacent hepatic curvature of the colon (Fig. 2a, b). We considered an infectious lesion (abscess) of the right kidney more likely than renal carcinoma and performed laparoscopic right nephrectomy, partial liver resection, and intestinal adhesion release.

### Gross and microscopic findings

**Fig. 3:**
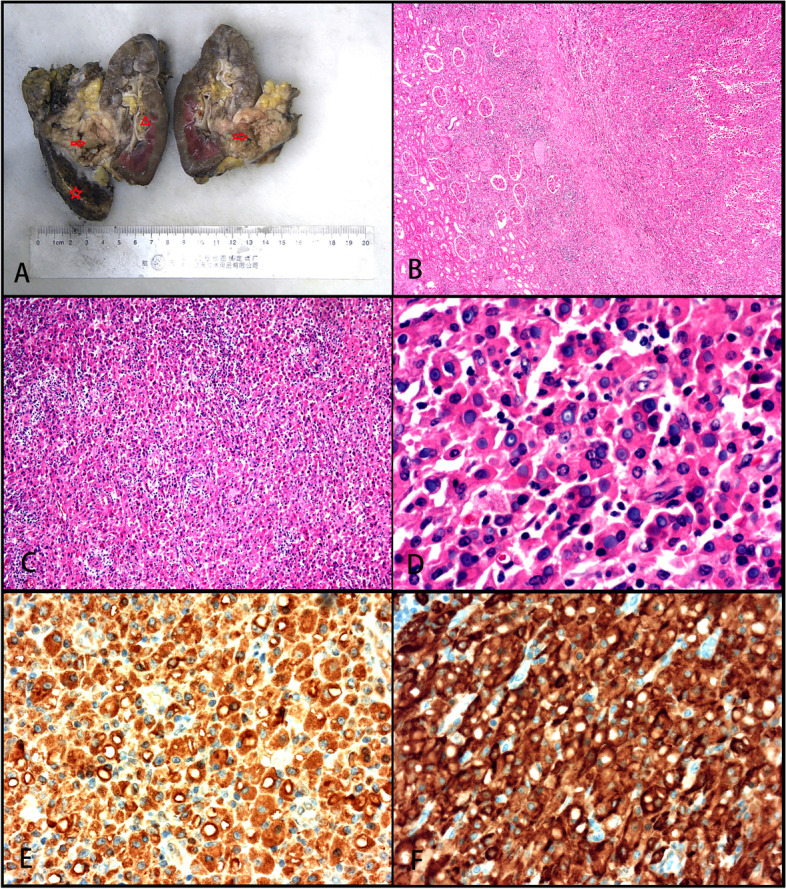
Gross and histological features of renal and liver malakoplakia. **A** A sallow, solid mass (red arrow) is evident in the kidney cortex (red triangle). It is invading the perirenal adipose tissue and is adherent to part of the liver (red star). **B** Clear boundary between the mass and the surrounding renal tissue, forming a thick fibrous pseudocapsule (H&E, magnification × 50). **C** Histocytes and von Hansemann cells distributed in sheets, with poor intercellular adhesion, abundant cytoplasm, eosinophilic and perivascular infiltration of lymphocytes, plasma cells, and neutrophils (H&E, magnification × 100). **D** Circular M-G bodies in the intracellular and extracellular interstitium, showing annular lamellar bodies with a bull’s eye or hawk’s eye appearance (H&E, magnification × 600). **E** Histiocytes showing diffuse cytoplasmic CD68 staining (magnification × 100). **F** Histiocytes showing diffuse cytoplasmic CD163 staining (magnification × 100)

A biopsy specimen (approximately 0.2 cm in diameter) was taken from the ascending colon. Surgical resection of the kidney (9.5 × 5 × 4.5 cm) attached to the ureter segment (2.5 cm in length and 0.6 cm in diameter) was performed. Incision of the kidney revealed a mass (4 × 3.6 × 3 cm) in the renal parenchyma 3 cm from the renal hilum. The section of the mass was sallow, solid, and soft. The mass had penetrated through the fibrous membrane of the kidney to invade the perirenal fat tissue and had adhered to part of the liver (Fig. 3a). Biopsy of the ascending colon revealed the disappearance of intestinal mucosal glands and mucosal tissue largely replaced by cells distributed in sheets. Neutrophil and lymphocyte infiltrates were seen around thin-walled blood vessels (Fig. 1b). The lesions in the renal parenchyma and hepatic parenchyma were well bounded (Fig. 3b), with thick incomplete fibrous envelopes and distributed sheets of histiocytes or von Hansemann cells, whose cytoplasm contained periodic acid-Schiff (PAS)-positive inclusion bodies resistant to amylase digestion. The adhesion between cells was poor (Fig. 3c). The cells were round and polygonal, the cytoplasm was rich, eosinophilic, and granular, and the nuclei were round and centered or eccentric, with 1–2 small nucleoli observed. The cell boundaries were not clear. The cells were focally short fusiform and arranged in bundles. M-G bodies were found in the intracellular and extracellular stroma with circular inclusion bodies of 1–10 μm, comparable to the size of tissue nuclei. M-G bodies were observed as annular lamellar bodies with a bull’s eye or hawk’s eye appearance (Fig. 3d). They were slightly gray in appearance, similar to the nuclei in hematoxylin and eosin (H&E)-stained sections. Histiocytes were mixed with lymphocytes, plasma cells, and neutrophils.

**Fig. 4:**
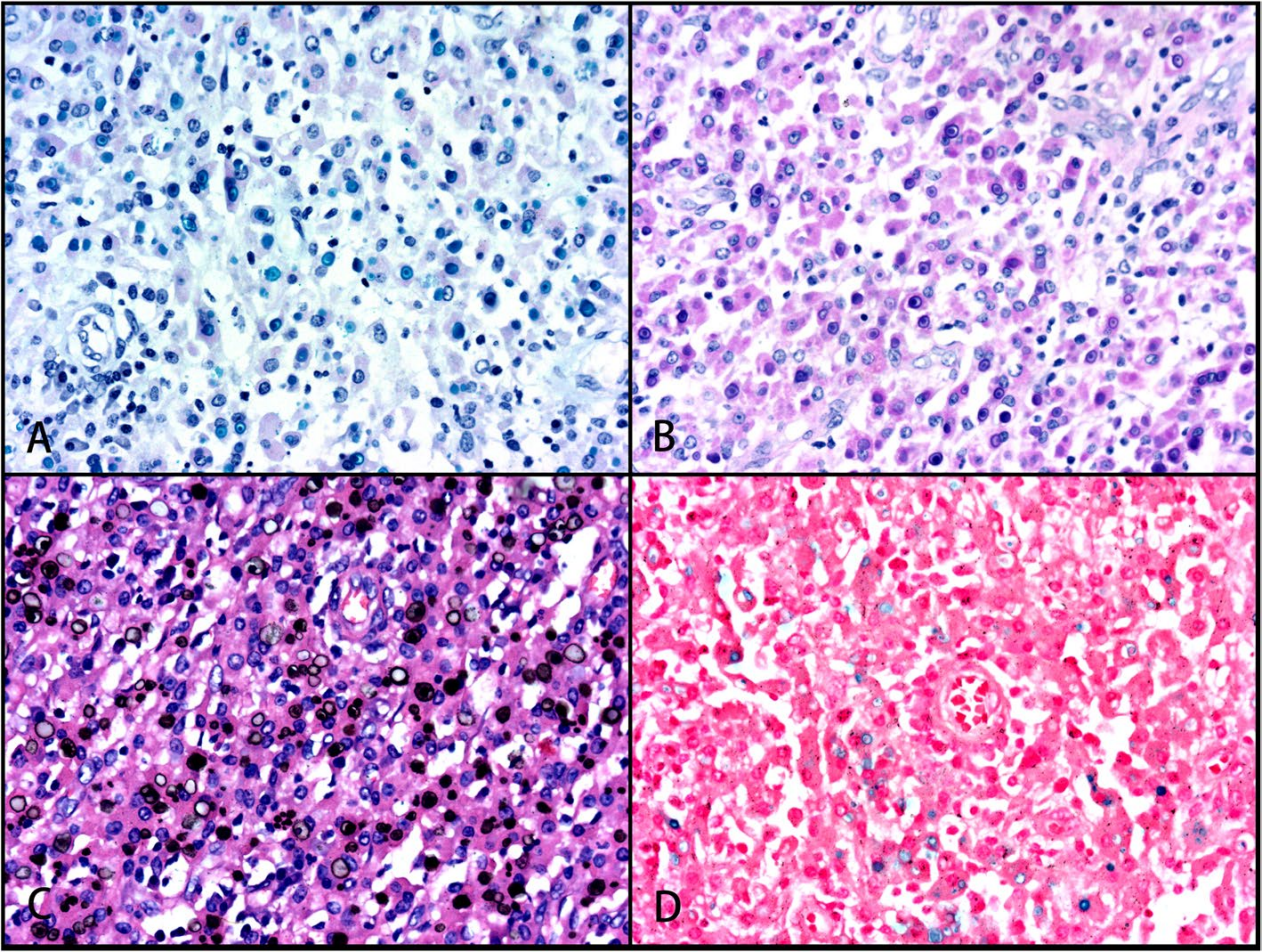
Malakoplakia special staining. **A** Blue M-G bodies on AB-PAS staining (magnification × 100). **B** PAS staining showing cytoplasmic red staining of histocytes and von Hansemann cells and purplish red M-G bodies (magnification × 100). **C** Black M-G bodies on von Kossa staining (magnification × 100). **D** Blue M-G bodies on Prussian blue staining (magnification × 100)

**Fig. 5:**
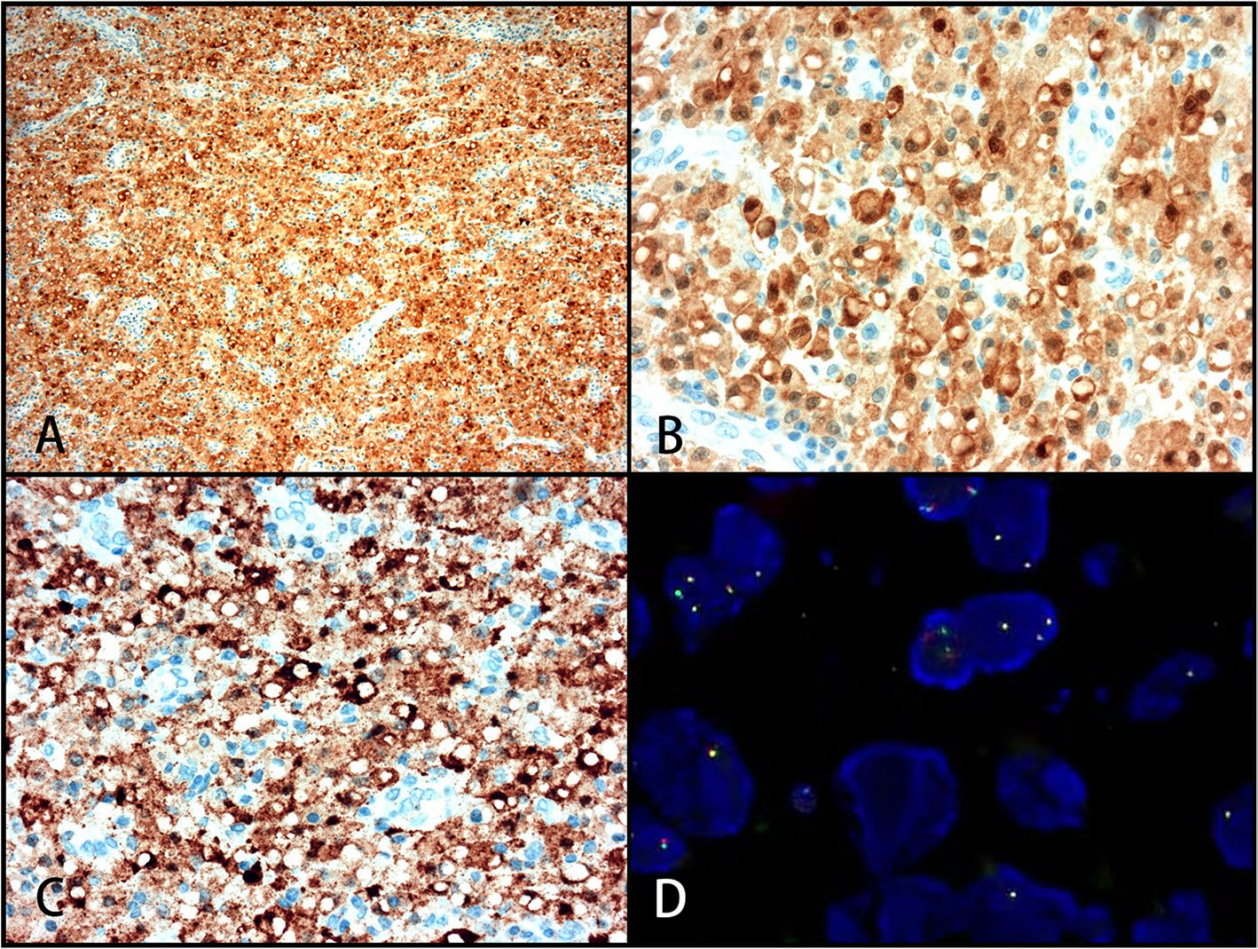
Malakoplakia ALK IHC staining. **A** Diffuse ALK1 staining (magnification × 100). **B** Cytoplasmic and nuclear ALK staining, but M-G bodies are not stained (magnification × 400). **C** Cytoplasmic and nuclear ALK (D5F3) staining, but M-G bodies are not stained (magnification × 400). **D** No break of the *ALK* gene detected on FISH (magnification × 400)

The cells were negative for CK, CD21, CD23, CD35, CD1α, S100, Langerin, and CD30, while the cytoplasmic granules were positive for CD10, CD68, and CD163 (Figs. 1c and d, 3e and f) as well as ALK1 and ALK (D5F3). The cytoplasm and nucleus of some cells were also positive (Fig. 5a–c), and the Ki-67 index was approximately 2%. PAS staining and amylase digestion showed PAS staining of histocytes and von Hansemann cells. M-G body corpuscular Alcian blue (AB) staining was blue (Fig. 4a), PAS and PAS with diastase staining was purple red (Fig. 4b), von Kossa staining was black (Fig. 4c), Prussian blue staining was blue (Fig. 4d), and PAS-methenamine silver staining was uncolored. The percentage of cells with a positive *ALK* gene break signal detected by fluorescence in situ hybridization (FISH) was 2%, indicating a negative result (Fig. 5d). Whole exome next-generation sequencing (NGS) revealed nine individual somatic gene mutations and zero germline mutations. The genes with somatic cell changes were: *GOT1L1, GLIS2, SPOUT1, TMEM97, MUC3A, NSD2, SFXN5, ADAD1* and *RAD50* (Table 1); no *ALK* gene mutation was detected.

**Table 1:** Mutations in nine individual genes detected by whole exome NGS

**Gene**	**Mutation type**	**Exon**	**Result of mutation**
*GOT1L1* NM_152413.3	Frameshift	9	c.1243dup p.T415Nfs*?
*GLIS2* NM_032575.2	Frameshift	6	c.1190dup p.M398Hfs*69
*SPOUT1* NM_016390.4	Frameshift	3	c.102dup p.W35Mfs*77
*TMEM97* NM_014573.3	Frameshift	3	c.528del p.K176Nfs*188
*MUC3A* NM_005960.2	Missense	2	c.1151_1152delinsCC p.M384T
*NSD2* NM_133330.2	Frameshift	24	c.4028dup p.E1344Rfs*91
*SFXN5* NM_144579.3	Missing in frame	1	c.36_38del p.A13del
*ADAD1* NM_139243.4	Frameshift	4	c.244del p.I82Yfs*6
*RAD50* NM_005732.4	Frameshift	13	c.2165dup p.E723Gfs*5

*indicates a mutation but no specific amino acid substitution. It usually indicates a stop codon or a nonsense mutation. A stop codon is a signal in protein synthesis that indicates that protein synthesis should stop

### Treatment course

Ultrasound-guided puncture biopsy of the right renal mass was performed, and pus was extracted by puncture drainage. Culture of the pus indicated the presence of *Enterococcus faecium* and *E. coli*. Blood culture indicated the presence of *E. faecium* and *Bacillus fragilis*. T cell subpopulation analysis revealed an increase of Th(CD3 + CD4 +)/Ts(CD3 + CD8 +) cells. Based on the results of drug sensitivity analysis, the antibiotics imipenem and vancomycin were administered. The patient was discharged after improvement, with no discomfort during 30 months of follow-up.

## Discussion and conclusions

Malakoplakia most commonly affects the urinary system but has also been reported in the gastrointestinal tract, skin, reproductive tract, lung, brain, lymph nodes, pancreas, and retroperitoneum. In the urinary system, it usually affects the bladder, especially in female patients [3], although lesions can also occur in the kidney and urethra. The second most common site is the gastrointestinal tract, usually affecting the rectum and colon [4]. Initially, malakoplakia appears as soft yellow to tan mucosal plaques and later as raised gray to tan lesions, varying in size with a central depression and surrounding redness [1]. Light yellow masses with medium texture and poorly defined boundaries can also form, and cystic changes can occur in the center of the masses [5].

Microscopically, malakoplakia is characterized by patches of lightly stained or slightly granular histocytes and von Hansemann cells, which are histocytes undergoing specific pathological changes. Von Hansemann cells are round or polygonal cells, and may, in rare cases, be fusiform [6]. Characteristic M-G bodies are seen both in the intracellular and extracellular interstitium, which are small spheroids that are formed from a mixture of calcium, iron, phosphorus, and organic matter. Certain stains including AB, PAS, von Kossa, and Prussian blue can clearly show M-G bodies. Electron microscopy indicates that the cells contain a large number of phagocytic lysosomes and layered crystals, and occasionally bacteria. Immunofluorescence analysis shows positive intracellular IgA, IgG, IgM, and light chain reactions. The cytoplasm is positive for CD10, CD68, and CD163 on immunohistochemistry (IHC).

The histological morphology of malakoplakia varies over time and can be divided into three stages. In the first stage, the number of cells is relatively small, and lymphocyte and plasma cell infiltration can be seen in the interstitium. This stage is considered to represent the glucolipid aggregation of M-G bodies. In the second stage, more histiocytes and von Hansemann cells appear, and a large number of M-G bodies form inside and outside the cells, which is the most characteristic and recognizable stage. In the third stage, the number of histocytes and von Hansemann cells gradually decreases, and interstitial fibrosis and collagen fiber hyperplasia occur at the terminal stage [7, 8].

The etiology and pathogenesis of malakoplakia are still not fully understood, but many mechanisms have been proposed [9]. Bacterial infections such as *E.coli* (accounting for 80% of cases), *Enterococcus sp.*, *Klebsiella pneumoniae*, *Pseudomonas aeruginosa*, *Staphylococcus aureus*, and other bacteria are involved in the pathogenesis of malakoplakia [7]. Impairment of the bactericidal activity of macrophages plays an important role in pathogenesis [1, 5]. It may be due to reduced cyclic guanosine monophosphate levels and beta glycosidase enzyme activity, which affects the aggregation of microtubules and leads to insufficient bacterial degradation in lysosomes, and the cells cannot release lysosomes. The intracellular lysosomes expand and merge, which is followed by degenerative changes in the matrix and limiting membrane. Incompletely digested bacteria then accumulate in lysozymes, forming characteristic concentric circles of M-G bodies with the deposition of calcium and iron salts [10]. Correction of lysosomal defects with cholinergic agonists is therapeutically beneficial [11]. In more than 50% of cases, malakoplakia occurs in patients with immunocompromised or chronic diseases [12], such as human immunodeficiency virus/acquired immune deficiency syndrome autoimmune diseases, diabetes, tuberculosis, myelodysplastic syndrome, tumors, and other diseases after transplantation [13]. However, malakoplakia is not as common in immunocompromised patients or patients with chronic disease, possibly because the bactericidal function of macrophages is retained. M-G bodies are a typical morphological feature of malakoplakia and can also be found in peripheral blood mononuclear cells, suggesting that the lesions are systemic [12].

ALK is a transmembrane tyrosine kinase belonging to the insulin receptor superfamily [14]. The *ALK* gene is highly conserved and is located on human chromosome 2p23 [15]. ALK-positive tumors include the following [16]: 1) lymphohematopoietic tumors, including anaplastic large cell lymphoma, ALK-positive large B-cell lymphoma, and ALK-positive histiocytosis; 2) epithelial tumors, including non-small cell lung cancer, ALK-positive renal cell carcinoma, and other types of cancer; and 3) mesenchymal tumors: including inflammatory myofibroblastic tumor, epithelioid inflammatory myofibroblastic sarcoma, epithelioid fibrous histiocytoma [17], and alveolar rhabdomyosarcoma and gastrointestinal leiomyoma [18].

Since malakoplakia can express ALK aberrantly, it needs to be differentiated from ALK-positive tumors, especially from ALK-positive histiocytosis [19]. ALK-positive histiocytosis is characterized by extensive histiocytosis with irregularly folded nuclei, fine chromatin, and abundant eosinophilic cytoplasm, sometimes with emperipolesis. Immunostaining showed that the histiocytes were positive for ALK, histiocytic markers (CD68, CD163) and variably S100, while being negative for CD1a, CD207, BRAF-V600E, and frequent presence of *KIF5B*::*ALK* gene fusion. The ALK inhibitor crizotinib is an effective treatment option. In the present case, although the malakoplakia was positive for ALK IHC, no *ALK* fusion gene was detected, and typical intracellular and extracellular M-G bodies were found (Table 2).

**Table 2:** Differential diagnosis of malakoplakia and ALK-positive histiocytosis

Tumor type	Malakoplakia	ALK-positive histiocytosis
Age of onset	Predominantly of adults	Predominantly of young age
Site of disease	Systemic or localized disease. Systemic disease involved multiple organs. Localized disease commonly affects urogenital tract and gastrointestinal tract.	Systemic or localized disease. Systemic disease tended to be young children, presented with hepatosplenomegaly, anemia and thrombocytopenia. Localized disease tended to be older, presented with tumors in the following sites: nasal skin, intracranial cavernous sinus, foot and breast.
Histologic features	Sheets of histiocytes with poor intercellular adhesion, with Michaelis–Gutmann bodies present in both the intracellular and extracellular interstitium.	Extensive histiocytosis with irregularly folded nuclei, fine chromatin, and abundant eosinophilic cytoplasm, sometimes with emperipolesis.
Immunohistochemistry	Rarely positive for ALK. Positive for histiocytic markers (CD68, CD163). Negative for CD1a, CD207, BRAF-V600E.	Always positive for ALK. Positive for histiocytic markers (CD68, CD163). Negative for CD1a, CD207, BRAF-V600E.
Molecular pathology	No *ALK* fusion gene.	Frequent presence of *KIF5B*::*ALK* gene fusion, a few cases presence of *COL1A2::ALK* gene fusion.
Treatment	The antibiotics is an effective treatment option.	The ALK inhibitor crizotinib is an effective treatment option.

Our patient had a history of diabetes for many years. Malakoplakia involved multiple organs, including the liver, kidney, and colon and was considered systemic. ALK showed a cytoplasmic diffuse granular-positive staining pattern, and some cells showed cytoplasmic and nuclear ALK staining at the same time, but no abnormality was found on *ALK* gene screening. NGS revealed nine individual somatic gene mutations: *GOT1L1, GLIS2, SPOUT1, TMEM97, MUC3A, NSD2, SFXN5, ADAD1* and *RAD50*. *GOT1L1* was reported to show L-aspartate aminotransferase activity and thus could be involved in the synthesis of D-aspartate, which serves as the agonist of N-methyl-D-aspartate receptor (NMDAR) [20]. *GLIS2* gene, which encodes a Kruppel-like zinc finger transcription factor. *GLIS2* plays an important role in maintaining normal kidney structure and function by preventing apoptosis and fibrosis, and its mutation is linked to tubule atrophy and progressive fibrosis [21]. *SPOUT1* is essential during the mitotic spindle of the metaphase [22]. Recent literature implicates *TMEM97* gene involvement in cholesterol homeostasis and Niemann-Pick disease [23]. *MUC3A* a main member of the mucin family, is commonly expressed on the surface of various epithelial cells, especially intestinal epithelial cells. Research shows that distinct variants of *MUC3A* may be involved in the occurrence of ulcerative colitis and Crohn’s disease [24]. *NSD2* is an epigenetic regulator for histone methylation in histone 3 lysine 36 (H3K36) [25]. *NSD2* is overexpressed, amplified or somatically mutated in multiple types of cancer, suggesting its critical role in cancer. The most well-known genetic alteration of *NSD2* has been t (4;14) translocation in multiple myeloma, which confers a poor prognosis [25]. *SFXN5* is primarily expressed in the brain [26]. ADAD1 is testis-specifc adenosine deaminase (AD) domain proteins, which is essential regulator of male germ cell [27]. The *RAD50, MRE11*, and *NBN* genes encode for the nuclear MRN protein complex, which senses the DNA double strand breaks and initiates the DNA repair [28]. In conclusion, the significance of the somatic gene mutations detected in this study is not clear, and the relationship between them and malakoplakia cannot be clarified by existing scientific studies.

This is the first case of malakoplakia with aberrant ALK expression to be reported, the aberrant expression of ALK in malakoplakia must be documented to avoid misdiagnosis with ALK-positive histiocytosis which is driven by an underlying gene fusion that can be targeted with small molecule tyrosine kinase agents.

### Supplementary Information

**Additional file 1.**

## Abbreviations

AB: Alcian blue
ALK: Anaplastic lymphoma kinase
FISH: Fluorescence in situ hybridization
IHC: Immunohistochemistry
H&E: Hematoxylin and eosin
M-G: Michaelis-Gutmann
NGS: Next-generation sequencing
PAS: Periodic acid-Schiff
