# Supplementary figures and images for: Malakoplakia with aberrant ALK expression by immunohistochemistry: a case report
Source: Diagn Pathol. 2023 Aug 29;18:97. doi: 10.1186/s13000-023-01383-z (PMC10464214; doi:10.1186/s13000-023-01383-z)

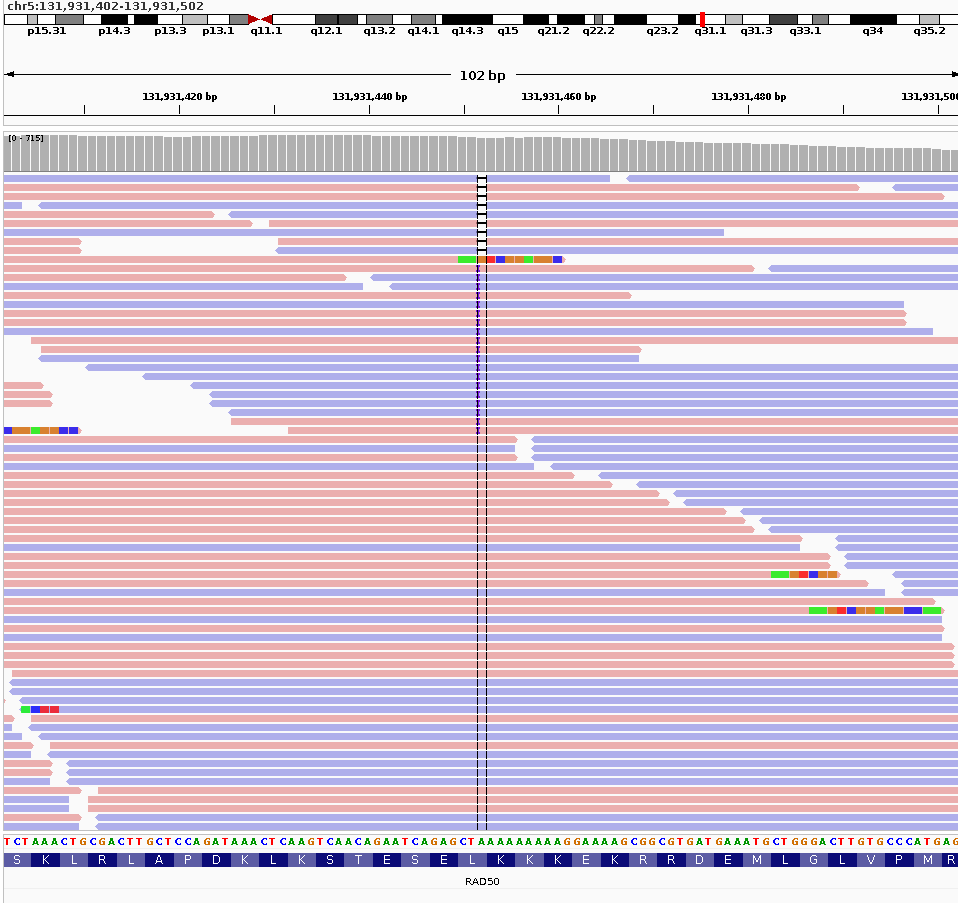

Supplement: Supplementary file 1 — Additional file 1. [file 13000_2023_1383_MOESM1_ESM.zip › 10-RAD50.png]

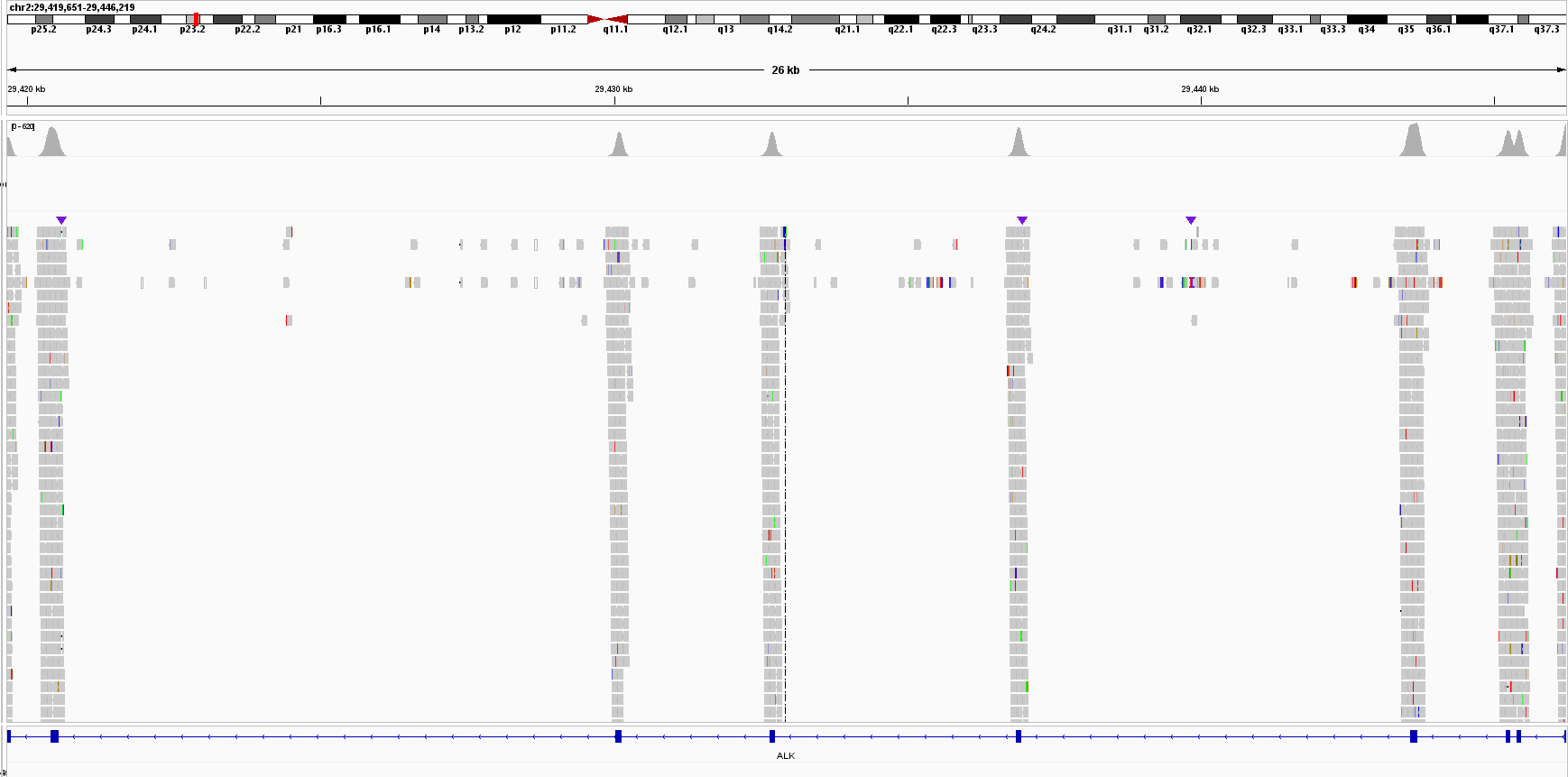

Supplement: Supplementary file 1 — Additional file 1. [file 13000_2023_1383_MOESM1_ESM.zip › 1-ALK.png]

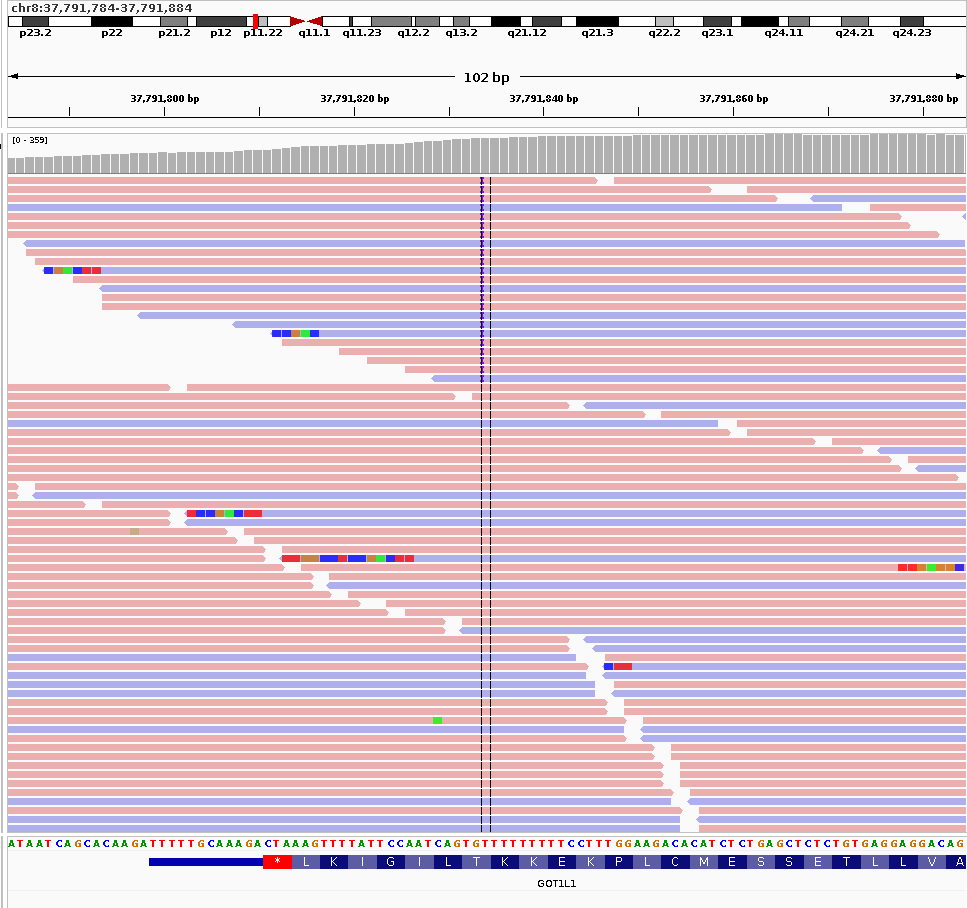

Supplement: Supplementary file 1 — Additional file 1. [file 13000_2023_1383_MOESM1_ESM.zip › 2-GOT1L1.png]

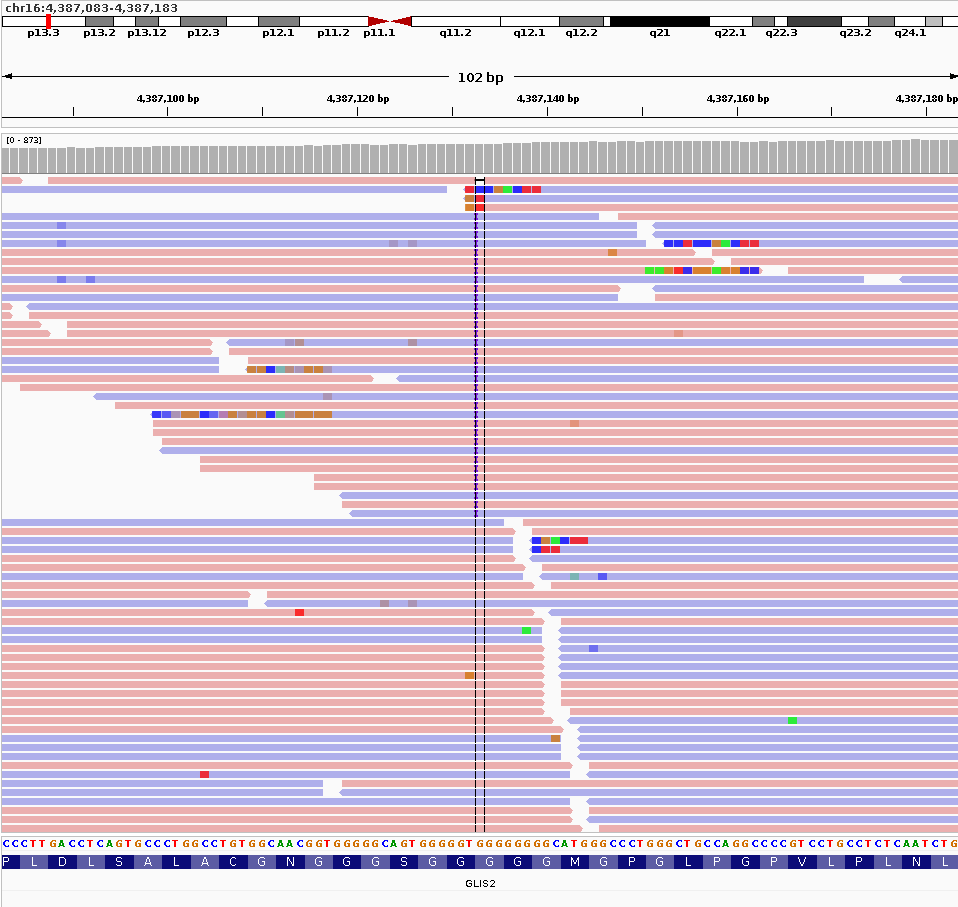

Supplement: Supplementary file 1 — Additional file 1. [file 13000_2023_1383_MOESM1_ESM.zip › 3-GLIS2.png]

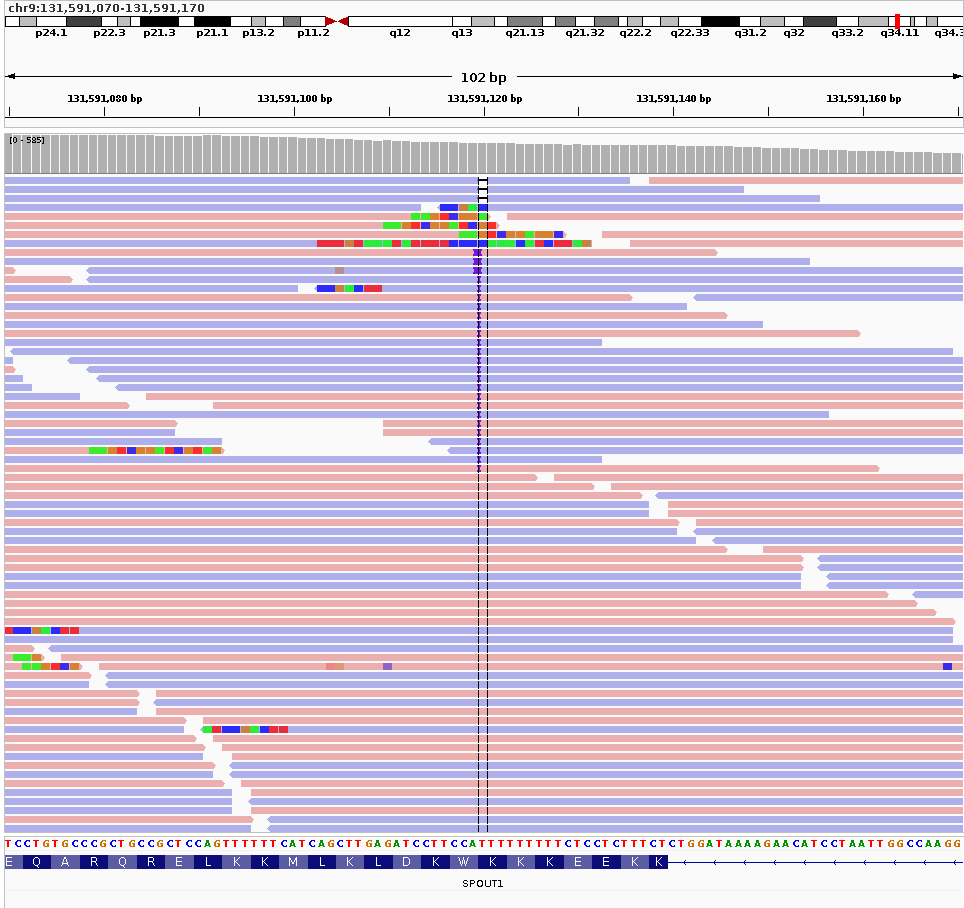

Supplement: Supplementary file 1 — Additional file 1. [file 13000_2023_1383_MOESM1_ESM.zip › 4-SPOUT1.png]

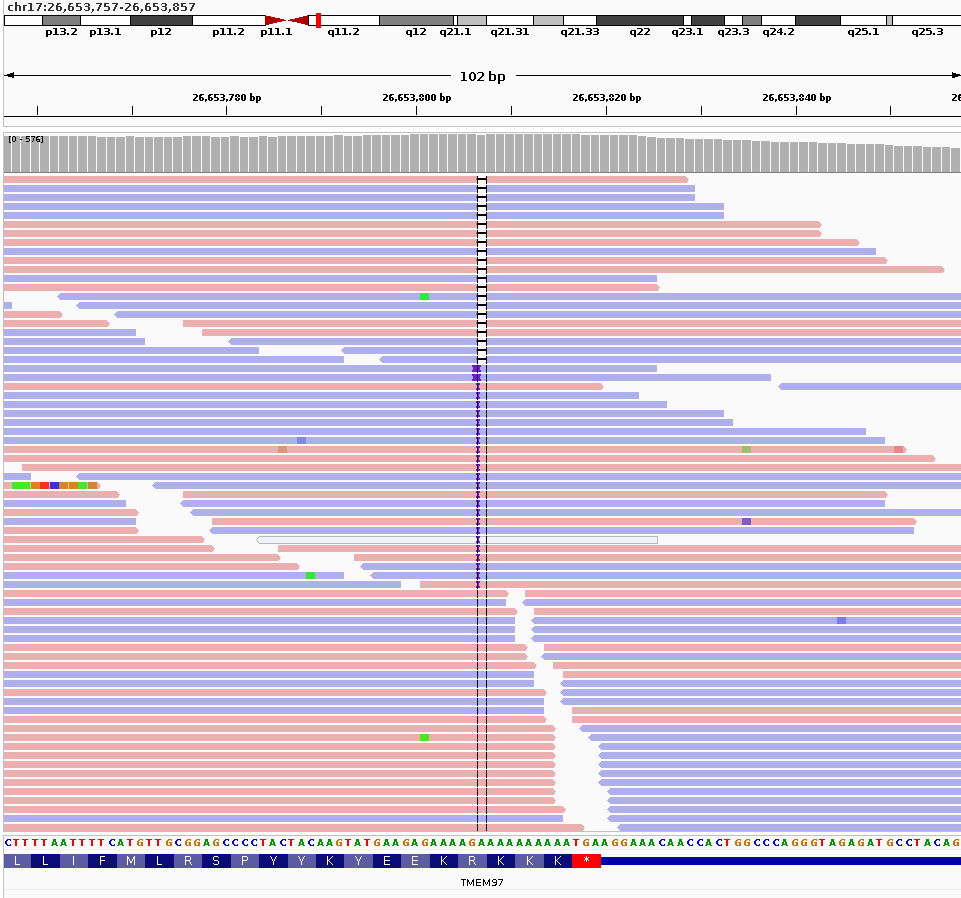

Supplement: Supplementary file 1 — Additional file 1. [file 13000_2023_1383_MOESM1_ESM.zip › 5-TMEM97.png]

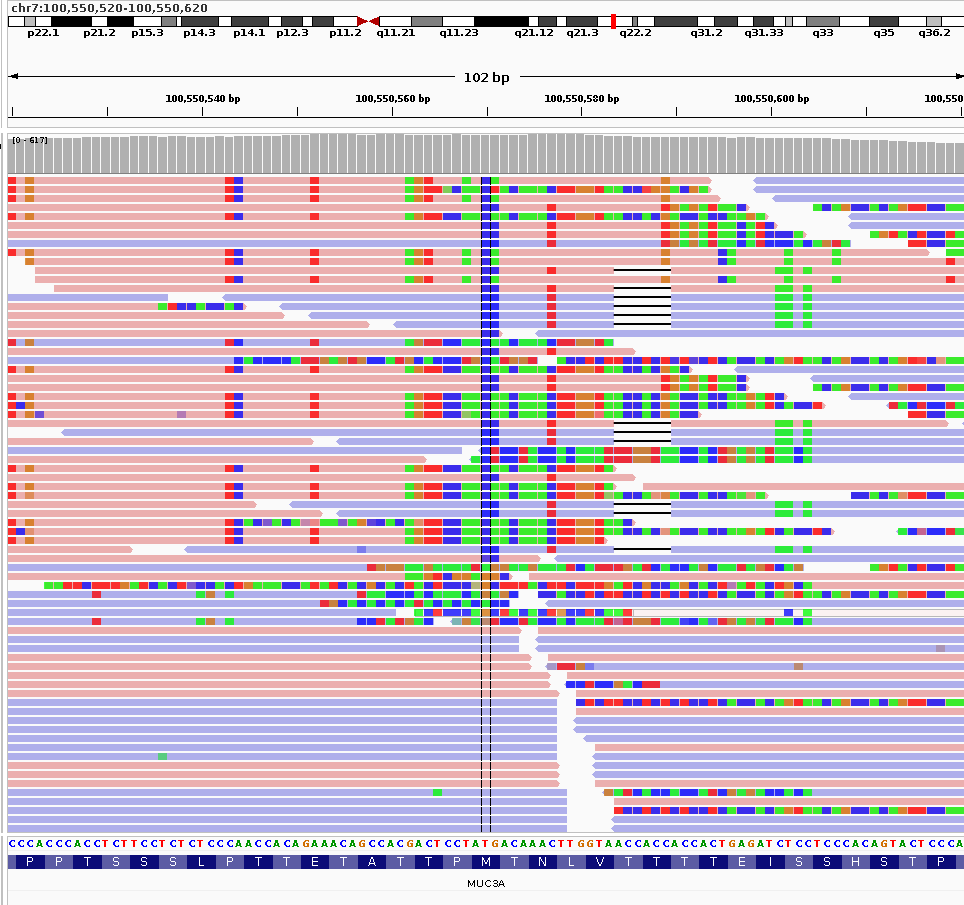

Supplement: Supplementary file 1 — Additional file 1. [file 13000_2023_1383_MOESM1_ESM.zip › 6-MUC3A.png]

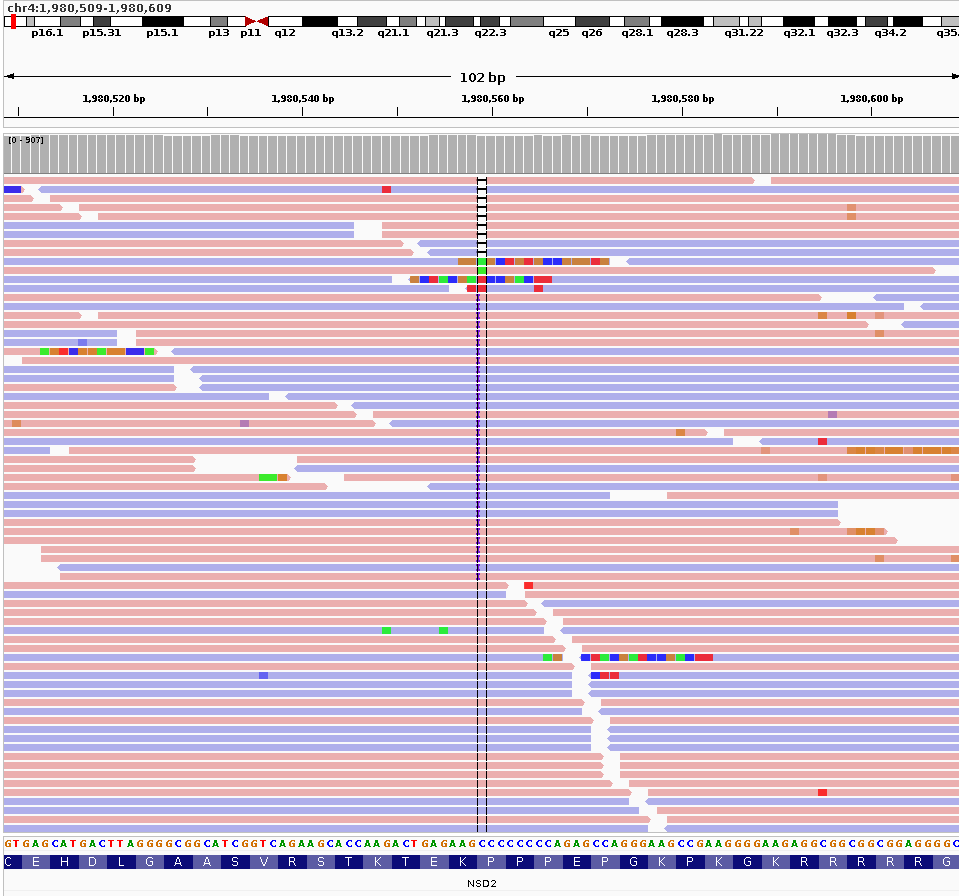

Supplement: Supplementary file 1 — Additional file 1. [file 13000_2023_1383_MOESM1_ESM.zip › 7-NSD2.png]

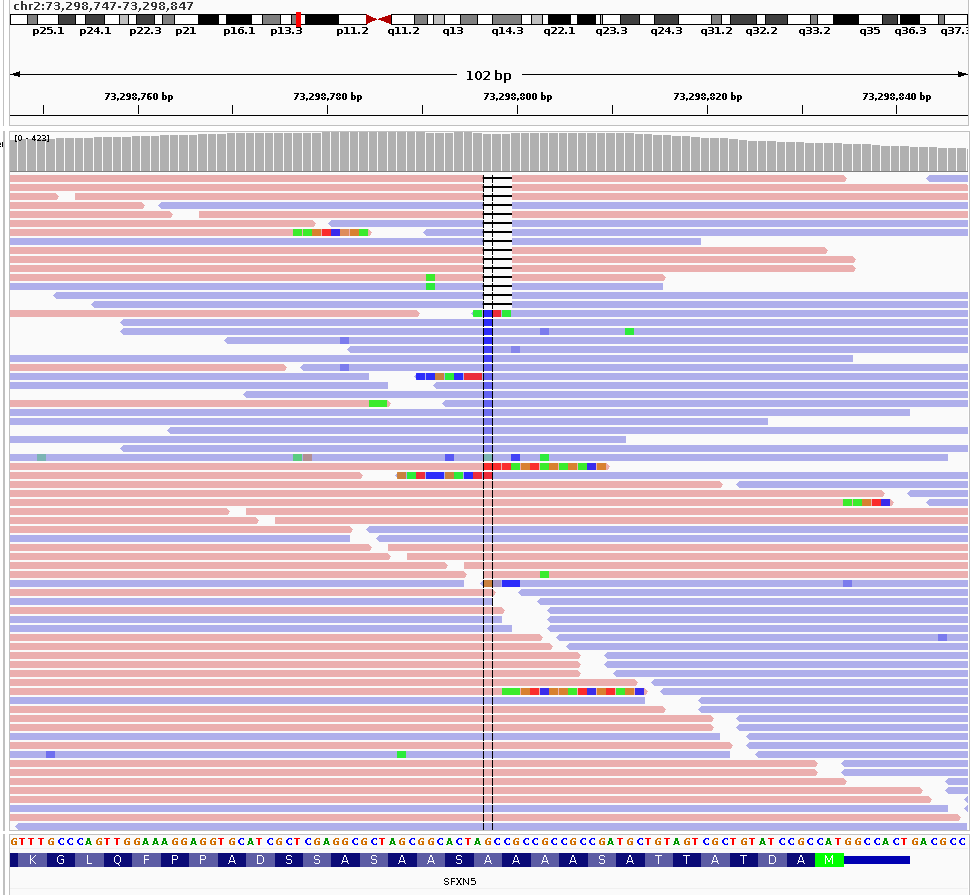

Supplement: Supplementary file 1 — Additional file 1. [file 13000_2023_1383_MOESM1_ESM.zip › 8-SFXN5.png]

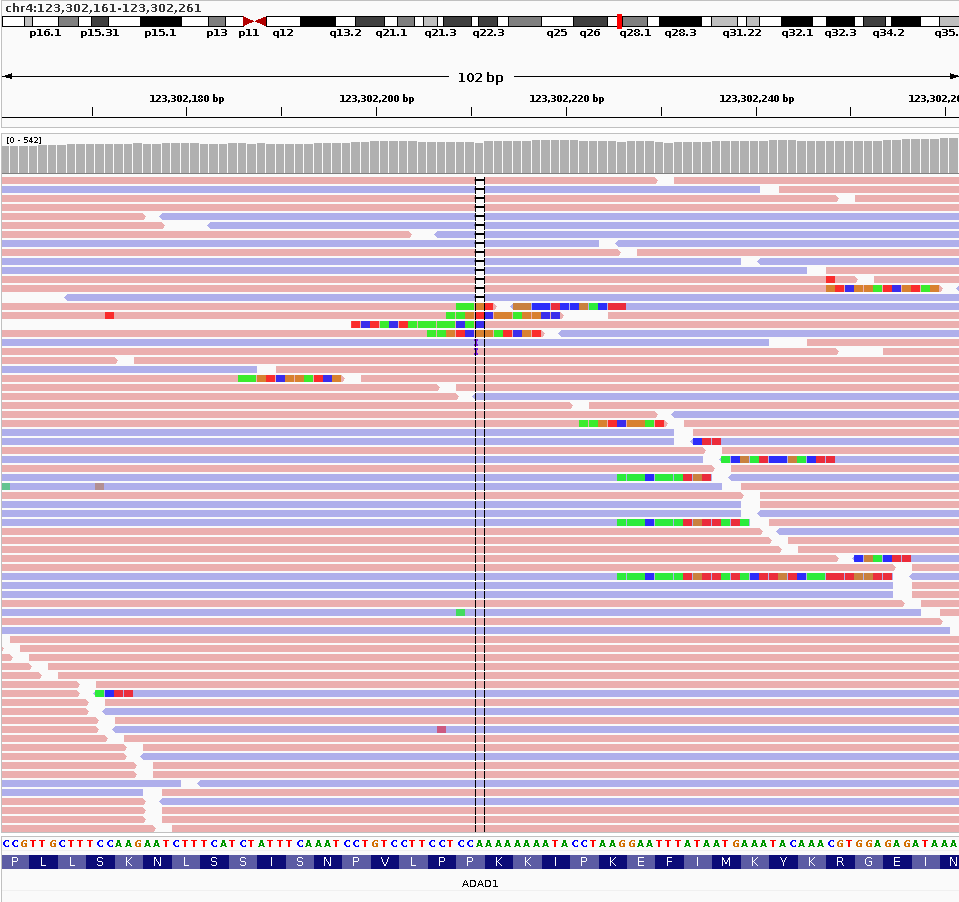

Supplement: Supplementary file 1 — Additional file 1. [file 13000_2023_1383_MOESM1_ESM.zip › 9-ADAD1.png]
